# Relationship Between Depression and Falls Among Nursing Home Residents: Protocol for an Integrative Review

**DOI:** 10.2196/46995

**Published:** 2023-10-19

**Authors:** Alcina Matos Queirós, Armin von Gunten, Joëlle Rosselet Amoussou, Maria Manuela Martins, Henk Verloo

**Affiliations:** 1 Department of Health and Social Welfare University of Lausanne Lausanne Switzerland; 2 Institute of Biomedical Sciences Abel Salazar University of Porto Porto Portugal; 3 Service of Old Age Psychiatry Lausanne University Hospital and University of Lausanne Lausanne Switzerland; 4 Medical Library-Cery Lausanne University Hospital and University of Lausanne Lausanne Switzerland; 5 School of Nursing Sciences HES-SO Valais-Wallis University of Applied Sciences and Arts Western Switzerland Sion Switzerland

**Keywords:** depression, falls, nursing homes, nursing home residents, older adults, fall risk, intervention

## Abstract

**Background:**

Aging exposes individuals to new health disorders and debilitating chronic diseases, yet most older adults, even in functional decline, do not want to leave their homes. Nevertheless, for many, institutionalization in a nursing home (NH) may become essential to ensure their continued safety and health. Depression is one of the most common psychiatric disorders among older adults, especially among those who are institutionalized. Depressed NH residents face a high risk of future functional decline and falls, decreasing their quality of life. The relationship between depression and falls is complex and bidirectional. Previous reviews have focused on home-dwelling older adults or explored the relationship between antidepressant drugs and falls. To the best of our knowledge, no integrative literature reviews have explored the relationship between depression and falls among NH residents.

**Objective:**

Analyze studies on the relationship between depression and falls among NH residents.

**Methods:**

We will conduct an integrative literature review of published articles in relevant scientific journals on the relationship between depression and depressive symptomatology and falls among NH residents. As usually defined, we will consider NH residents to be people aged 65 years and older who can no longer live safely and independently in their homes. We will also consider older adults on short-term stays in an NH for rehabilitation after hospital discharge. Retrieved articles will be screened for eligibility and analyzed following previously reported steps. The most pertinent bibliographical databases will be examined for qualitative, quantitative, and mixed methods studies, from inception until August 31, 2023, thus ensuring that all relevant literature is included. We will also hand-search the bibliographies of all the relevant articles found and search for unpublished studies in any language. If appropriate, we will consider conducting a meta-analysis of the studies retrieved.

**Results:**

A first round of data collection was completed in March 2023. We retrieved a total of 2276 references. A supplementary literature search to ensure the most up-to-date evidence is ongoing. We anticipate that the review will be completed in late September 2023, and we expect to publish results at the end of December 2023.

**Conclusions:**

This integrative review will increase knowledge and understanding of the complex relationship between depression and falls in NH environments. Its findings will be important for developing integrated, multidisciplinary models and care recommendations, adaptable to each NH resident’s situation and health status, and for creating preventive interventions to help them maintain or recover optimal health stability.

**International Registered Report Identifier (IRRID):**

DERR1-10.2196/46995

## Introduction

### Overview

The world’s population is aging fast. The World Health Organization predicts that about 2 billion people will be aged 65 years or older in 2030 [1-3]. For various biological, psychological, and societal reasons, older adults are at greater risk of developing psychopathologies such as depression and dementia, conditions which lead to further loss of autonomy and functional decline [4,5]. A recent study estimated a prevalence of depression of 7% among community-dwelling older adults [6]. A depressive episode can be defined as a mood disorder (a common mental and behavioral disorder) that persists for at least 2 weeks [7]. Depression can present at different levels of clinical severity—mild, moderate, or severe—and can occur on 1 unique occasion during a life course or be recurring or chronic [7]. Older adults enduring severe depression and other associated psychiatric diseases face a significant risk of planned or unplanned admission to a nursing home (NH) [8,9]. Several studies have reported that planned and unplanned institutionalization in an NH is often associated with debilitating medical comorbidities, a reduced ability to perform the activities of daily living, problems with mobility, and cognitive impairment [9]. NH residents, mostly older adults, are defined as people aged 65 years and older [10]. However, it is not easy to apply a strict definition because people age biologically at different rates, so, for example, someone aged 75 may be healthier than someone aged 65 [11]. NHs are facilities that provide 24-hour functional support for older adults who require assistance with the activities of daily living or the instrumental activities of daily living and have recognized health needs. They are staffed with health care professionals and provide long-term care and rehabilitation as part of hospital avoidance or to facilitate early hospital discharge; they do not function as hospital wards and are not hospital-based, and they may play a role in providing palliative and hospice care at the end of life [12]. We will also accept studies examining older adults staying in NHs for short-term rehabilitation after hospital discharge. However, most NH residents live there permanently because they have ongoing physical or mental conditions that require constant care and supervision [12].

The reported prevalence of depression among NH residents varies from 15%-48%, with variations caused by the diversity in methodological approaches and measurement instruments used [6,13-15]. Depressed NH residents often become less able to function, and the condition sometimes speeds them toward functional disability, with an increased risk of falls [16]. NHs implement person-centered care as best as they can, considering individuals’ needs and striving to avoid functional and mental decline (including disease- and drug-related fall risks) to ensure the best possible level of overall physical, emotional, and mental well-being for their residents [17,18]. However, most care is aimed at residents’ somatic complaints: even though psychiatric disorders are prevalent, particularly clinical depression, they frequently remain unidentified by health care staff [19,20]. Furthermore, concerning functional decline, it seems that falls are one of the most significant associated dangers faced by NH residents [21-25]. Multiple causes can contribute to heightening the risk of falls, including frailty, psychopathological disorders, and their pharmacological treatments—all of which are common among NH residents [26-28]. They can even include an NH resident’s new, spacious surroundings, involving more distance to cover along corridors or less furniture to hold onto [29-31]. However, with any of these factors, a predicted higher or lower risk may be underestimated or overestimated, creating the false reassurance that something is being done about falls. Falls often have severe consequences for NH residents, and between half and three-quarters of them may take a fall in any given year. Recent research reported that 10%-20% of falls result in serious injury, and a significant fraction of NH residents die from fall-related injuries [32]. Numerous factors can contribute to falls [31,33]. (1) Many NH residents are prescribed antipsychotics, antidepressants, Z-drugs, benzodiazepines, and other drugs to abate neuropsychiatric symptomatology; however, these substances can cause confusion, unsteady gait, and loss of balance. (2) Inadequate or insufficient NH staffing, especially at night, can cause problems when residents need to get up but no one is immediately available to help them. (3) An absence of sufficient adaptive equipment for mobility-impaired NH residents. (4) Existing health conditions, such as orthostatic hypotension, Parkinson disease, Alzheimer disease, and other neuropsychiatric diseases, can result in an unsteady gait that leads to falls. (5) An absence of any comprehensive fall prevention plan for mobility-impaired NH residents at risk of falls.

Depression and falls have a significant bidirectional relationship [34]. Excessive fear of falling, which is frequently associated with depression, increases the risk of falls [35,36]. Both depression and fear of falling are associated with impaired gait and balance, an association that is mediated through cognitive, sensory, and motor pathways. Our integrative review will explore the relationship between depression and falls. The relationship between the variables will determine whether the right conclusions are reached. In our review, a relationship is defined as the existence of a correspondence between the 2 variables of depression and falls—it is a complex and bidirectional relationship. The relationship between depression and falls could be positive or negative associations, symmetrical, causal, linear, or nonlinear [37]. We will also consider these relationships according to their nature or pattern. A relationship suggests that as 1 variable changes, the other also tends to change. For example, 2 variables may have a quadratic relationship. Moreover, depression will be considered as an independent risk factor for falls. Previous studies of prospective cohorts have shown that depression increases the risk of future falls. A recent meta-analysis summarized the findings of 17 prospective studies and found an odds ratio for the association of depression with falls of 1.63 (95% CI 1.36-1.94) [37]. Retrospective studies have reported 4 determinants of falls: postural sway, history of falls, handgrip strength, and depressive symptoms [38,39]. Symptoms of depression may have individual direct roles in promoting falls. Poor appetite and weight loss are commonly seen in geriatric depression, and nutritional deficiencies in vitamin D and folate may be related to falls [39]. The relationships between depression, cognitive performance, motor performance, and the risk of falls were illustrated by a recent study that found that the association between depression and slowed-choice stepping reaction time was mediated by 2 variables—quadriceps strength and executive function—that influenced choice stepping reaction time via simple reaction time and balance [40,41]. Depression and falls are also linked indirectly through several common risk factors. Functional decline, history of falls, and cognitive impairment has each been linked separately to both depression and falls. Poor physical health is also a notable risk factor for depression and falls. Vascular disease, in particular, and its related burden of white matter lesions, may produce concurrent changes in balance, gait, and mood [40]. The interaction between depression and falls may also be self-perpetuating. Depressive symptoms are particularly high among recurrent fallers, which may be related to the demoralizing effect of repeated falling [42]. Prospective cohort studies have reported that increased depressive symptoms were associated with increased falls [43]. Conversely, a lower rate of falls was associated with improved morale scores over the follow-up period. The depressive cognitive-affective symptoms seen in repeat fallers include a lower sense of self-efficacy and negative expectations of the future. Restricted activity and decreased social participation can be a complication of recurrent falls: the resulting social isolation is known to be a significant risk factor for depression among older adults [44].

Managing depression in fall-prone individuals is challenging since antidepressant medications can increase the risk of falls, selective serotonin reuptake inhibitors may increase the risk of fragility fractures, and data are lacking about the effect of fall rehabilitation programs on clinically significant depression [45]. Antidepressant medications are indicated to treat moderate to severe depression in fall-prone individuals, but this should include appropriate precautions, including a low starting dose and slow dose titration, the use of psychotropic monotherapy whenever possible, and monitoring for orthostatic hypotension [45-47]. Treating depressed NH residents at risk of falls is a major concern in those settings and deserves our full attention. There is a need to expand current knowledge about the potential relationship between falls and depression among NH residents.

### Review Aims and Objectives

This review sought to analyze the literature examining the relationship between depression or depressive symptomatology and falls among NH residents.

### Research Question

This integrative review will explore the following question: “What is the relationship between depression and falls among nursing home residents?”

## Methods

### Design

This integrative literature review aims to synthesize the literature reporting on the relationships between depression or depressive symptomatology and falls among NH residents receiving or not receiving treatment with antidepressant medication. The review will be conducted based on the guidelines contained in Coleen’s *Step-by-Step Guide to Conducting an Integrative Review* [48] in the following order: (1) formulation of a review question, (2) systematic literature search, (3) critical appraisal of the research retained, (4) literature analysis and synthesis, (5) discussion on new knowledge, and (6) preparing a dissemination plan for findings [49].

### Types of Participants

The review will consider studies focusing on older adults with a mean or median age of 65 years or older living in a geriatric or psychiatric NH.

### Eligibility Criteria

Textbox 1 presents the eligibility criteria for the types of information that publications retained in this integrative review should contain. These criteria were based on an exploratory literature review of sociodemographic features, long-term care facilities and rates of depression among NH residents. We also included NH residents’ contextual and empirical experiences. This review will consider NH’s skilled nursing facilities and long-term care facilities as synonyms, providing a wide range of health and personal care services.

A patient fall is defined as an unplanned descent to the floor, with or without injury [50]. The review will not consider publications reporting on the nonfatal falls of residents receiving antidepressant medication without a diagnosis of depression.

We will include mixed methods studies and use the Mixed Methods Appraisal Tool scale to assess their quality and bias [51], as reported on the Equator Network website.

**Inclusion and exclusion criteria for the population, phenomena of interest, type of publication, health care setting, and language.**

**Inclusion criteria**

- Population: Older adults (65 years or older) living in nursing homes
- Depression criteria: Depression (diagnosed by a physician) and depressive symptomatology (reported by health care professionals using validated scales), whether treated or not treated with antidepressants
- Falls: Incidence, prevalence, or occurrence of falls; recurrent falls; and reported or assessed risk of falls
- Setting: Long-term geriatric or psychiatric nursing home o nursing home
- Article types: Original prospective research studies with a descriptive, correlational, or cohort design; retrospective cohort studies; and mixed-methods studies
- Language: No restrictions

**Exclusion criteria**

- Population: Adults younger than 65 years
- Depression criteria: Nursing home residents prescribed antidepressant medication but without recognized depression or depressive symptomatology
- Falls: Near falls (not reported in most publications)
- Setting: Intrahospital or private nursing home contexts (older adults’ houses or apartments, assisted living apartments, and community care living)
- Article types: Meeting abstracts; conference abstracts; posters; guidelines, commentaries, editorials, opinion papers, and book reviews and case reports; literature reviews, whether narrative, rapid, scoping, or meta-syntheses, and systematic and meta-analyses

### Types of Studies

This integrative review will include studies addressing depressive conditions or symptomatology and may include other types of neuropsychiatric symptomatology. Studies examining concepts surrounding falls and falling will also be associated with the literature search strategy to respond to the research question. Eligible studies will include prospective research studies with a descriptive, correlational or cohort design, retrospective cohort studies, and mixed methods studies.

### Types of Sources

The integrative review’s searches will consider original articles to identify the prevalence of falls among NH residents and their relationships with depression or depressive symptomatology. We will consider publications in any language but will focus on those identified in languages that the research team masters. In cases where the study language is not mastered, we will contact the authors and ask them to complete this study’s data extraction and quality assessment forms. The articles retrieved have been screened, and the review was completed in August 2023.

### Search Strategy

The search strategy is developed in collaboration with a medical librarian (JRA). The following bibliographic databases will be examined for qualitative, quantitative, and mixed methods studies to ensure that all the relevant literature is included: Ovid MEDLINE ALL, Embase, CINAHL with Full Text, APA PsycInfo Ovid, Web of Science Core Collection, ProQuest Dissertations & Theses A&I, and Cochrane Library. All searches will be conducted without language or date restrictions. Textbox 2 presents an example of the equation developed in Embase.

**Example of the Embase search strategy.**

('nursing home'/exp OR 'nursing home patient'/exp OR 'long term care'/de OR 'assisted living facility'/exp OR 'home for the aged'/exp OR ((nursing NEXT/2 home*) OR “skilled nursing facilit*” OR (“long term” NEXT/3 care) OR “care home*” OR “assisted living facilit*” OR (“assisted living” NEAR/2 resident*) OR “extended care facilit*” OR “intermediate care facilit*” OR “medical home*” OR (institutionali* NEXT/2 elderly) OR “geriatric homes” OR “home for the elderly” OR “homes for the elderly” OR “home for the aged” OR “homes for the aged”):ab,ti,kw) AND ('aged'/exp OR 'elderly care'/de OR 'geriatric care'/exp OR 'geriatric patient'/de OR 'geriatrics'/exp OR 'home for the aged'/exp OR (elder* OR eldest OR geriatr* OR “old age*” OR (older NEXT/1 (patient* OR people OR subject* OR age* OR adult* OR man OR men OR woman OR women OR population* OR person*)) OR aging OR ageing OR senior* OR “late life” OR “oldest old*” OR “very old*” OR “home for the aged” OR “homes for the aged” OR geronto* OR psychoger*)) AND ('falling'/exp OR 'fear of falling'/exp OR 'fall risk'/exp OR (fall OR falls OR falling):ab,ti,kw) AND ('mood disorder'/de OR 'depression'/exp OR 'antidepressant agent'/exp OR (depress* OR “mood disorder*” OR “affective disorder*” OR “mood decline” OR antidepress*):ab,ti,kw) NOT [conference abstract]/lim

### Data Management

#### Selection Process

All the titles and abstracts identified in the searches will be independently screened by 2 reviewers (AMQ and HV) to assess which studies met the inclusion criteria. Disagreements will be resolved through discussions, and in cases where no agreement could be found, a consensus was reached after discussions with the coauthors (AvG and MMM). In addition, the reviewers will independently assess the full-text articles to see if they met the literature review’s inclusion criteria. Again, the coauthors discussed and resolved disagreements (HV, AvG, and MMM). The integrative review selection process flowchart will be drawn in accordance with the guidelines in the 2020 PRISMA (Preferred Reporting Items for Systematic Reviews and Meta-Analyses) statement [52] to present the number of articles identified at each step of the review selection process.

#### Data Extraction

Data extraction will use a specially designed and structured data extraction form. The following information will be collected from each study finally included in the review: (1) study authors, year of publication, study duration, and country location; (2) study characteristics, including setting, design, and sample size; (3) participants’ characteristics, including sex and mean (SD) age; (4) types of results, such as the prevalence of depression or depressive symptoms, depression evaluation tools, the prevalence of antidepressant use, and the prevalence of falls; (5) statistical results; and (6) the studies’ recommendations.

#### Assessment of the Risks of Bias in the Studies Included

The risks of bias in all the cross-sectional, retrospective, and prospective cohort studies; mixed methods studies; and pragmatic or randomized and nonrandomized controlled trials will be independently assessed by 2 reviewers (AMQ and HV). Disagreements will be resolved through discussion and consultation with coauthors (MMM and AvG). We will use the validated Robins-I tool for assessing the risk of bias in nonrandomized studies of interventions [53]: (1) preintervention and at intervention (bias due to confounding, bias in the selection of study participants, and bias in the classification of the intervention), and (2) postintervention (bias due to deviations from intended interventions, bias due to missing data, bias in the measurement of outcomes, and bias in the selection of the reported results) [53]. Bias in cohort and case-control studies will be assessed using the Newcastle-Ottawa Quality Assessment Form for Cohort Studies (NOSGEN) [54] and the Appraisal tool for Cross-Sectional Studies (AXIS) [55]. The NOSGEN tool covers two dimensions of bias and seven domains through which that bias might be introduced into nonrandomized studies of intervention: (1) preintervention and at intervention (bias due to confounding, bias in the intervention, bias due to deviations from intended interventions, bias due to missing data, bias in the measurement of outcomes, and bias in the selection of the reported results) [54]. The AXIS tool assesses study quality and risk of bias, but it does not provide a numerical scale for assessing that quality—a degree of subjective assessment is required [55]. How the tool is used thus has implications for interpreting the results as there will be differences in individuals’ judgments. However, it has been argued that numerical scales for quality can be problematic as the outputs from assessment checklists are not linear and, as such, difficult to sum up or weigh, making them unpredictable for assessing study quality.

Our search strategy will include being very careful to select original research papers only, and it will try to avoid duplicates of published data reappearing in longitudinal studies. Additionally, our data extraction sheet will pay special attention to longitudinal cohort studies and any secondary analyses of published results.

### Statistical Analyses

We will compute descriptive statistics of the review population’s mean and median age, the distribution of men and women, and the number of falls recorded in the studies retained. Additional descriptive statistics will be computed to report differences between NH residents’ profiles and types of NHs.

We will consider whether it is appropriate to combine the numerical results from all the studies retained—or at least 2 of them—in a meta-analysis to yield overall statistics (together with their CIs) summarizing the relationship between depression and falls based on risk ratios. For dichotomous outcomes, average intervention effects will be calculated as relative risks or odds ratio with 95% CIs using a random effects model [56]. A random effects model will also be used on continuous data to calculate weighted mean differences with 95% CIs. If required, we will calculate SDs from the SEs or 95% CIs presented in the articles. Heterogeneity will be quantified using the *I*^2^ and chi-square tests. Funnel plots will be drawn, and Egger tests will be computed to explore the possibilities of publication bias [57]. Reasons for effect estimate heterogeneity will also be sought via meta-analyses [58]. To explore the possible determinants of heterogeneity, we will conduct subgroup analyses according to selected study characteristics (eg, participants’ age and sex). Furthermore, sensitivity analyses will be calculated by (1) excluding relatively small studies (with fewer than 20 participants) and (2) restricting analyses to the best-quality studies. These data will also be analyzed using SPSS (version 29.0; IBM Corp).

## Results

A first round of data collection was completed in March 2023. Our search in Ovid MEDLINE ALL (n=385), Embase (n=810), Cinahl with full text (n=303), APA PsycINFO Ovid (n=178), Cochrane Library (n=123), Web of Science (n=455), and ProQuest Dissertations and Theses A&I (n=22) returned 2276 references. A supplementary literature search to ensure the most up-to-date evidence is ongoing. Studies will be screened independently by 2 research team members. The inclusion and exclusion criteria will be rigorously respected throughout the study selection. Disagreements in article selection will be resolved through discussion. We anticipate that the review will be completed in late September 2023, and we expect to publish results at the end of December 2023.

## Discussion

### Principal Findings

This literature review will investigate original work on the sometimes complex and bidirectional relationship between depression or depressive symptomatology and falls or fall risks among NH residents. Depression will be considered an independent risk factor for falls, one that may lead to increased fall risks through behavioral, neuromuscular, or psychopathological pathways, as demonstrated in prospective cohort studies [59-61]. We will retrieve studies reporting on depression and falls identified as risks among NH residents, mostly presenting poor physical and mental health and producing concurrent changes in balance, gait, and mood. The review will explore studies indicating any evidence of the bidirectional relationship between depression and falls due to deficits in executive function, poor nutritional status, and cognitive disorders. Factors related to falls will be highlighted if they are documented in the publications retrieved. Although the risk factors identified do not always contribute directly to falls, our review will assess whether these factors are useful for detecting fall risks among NH residents.

### Limitations of the Literature Review

Although antidepressant medication can mitigate depression or depressive symptoms, which should lower fall risks, it can also increase fall risks independently of depression. We will not consider the contributing factor of antidepressant medication as an independent risk factor for falls within the relationship between falls and depression.

### Conclusions

The researchers anticipate that this integrative literature review will increase overall knowledge and understanding of the complexity of the phenomena of depression and falls in NH environments. Indeed, most preceding reviews have focused on home-dwelling older adults or research into antidepressant drugs and fall. The proposed study’s findings will be important for developing integrated, multidisciplinary models and recommendations of care that can be adapted to each NH resident’s situation and health status, and for creating preventive interventions to enable them to maintain or recover optimal health stability.

## Abbreviations

AXIS: Appraisal tool for Cross-Sectional Studies
NH: nursing home
NOSGEN: Newcastle-Ottawa Quality Assessment Form for Cohort Studies
PRISMA: Preferred Reporting Items for Systematic Reviews and Meta-Analyses

## Data Availability

The data sets generated during this study are available from the corresponding author on reasonable request.

## References

[1] (2012). Encyclopédie statistique de la Suisse. Office Fédéral de la Statistique.

[2] (2013). Organisation de coopération et de développement économiques. Wikipedia.

[3] (2021). United Nations Demographic Yearbook 2019. United Nations.

[4] (2019). Vieillir en bonne santé: Aperçu et perspectives pour la Suisse. Office fédéral de la santé publique.

[5] Kingston A, Wohland P, Wittenberg R, Robinson L, Brayne C, Matthews FE, Jagger C, Cognitive FunctionAgeing Studies collaboration (2017). Is late-life dependency increasing or not? A comparison of the Cognitive Function and Ageing Studies (CFAS). Lancet.

[6] Chun A, Reinhardt JP, Ramirez M, Ellis JM, Silver S, Burack O, Eimicke JP, Cimarolli V, Teresi JA (2017). Depression recognition and capacity for self-report among ethnically diverse nursing homes residents: Evidence of disparities in screening. J Clin Nurs.

[7] Sekhon S, Gupta V (2020). Mood Disorder.

[8] Höglinger M, Seiler S, Ehrler F, Maurer J (2019). Gesundheit der älteren Bevölkerung in der Schweiz. Bundesamt für Gesundheit BAG.

[9] Diebold M, Widmer M, Observatoire suisse de la santé (2019). Indicateurs de la santé de la population âgée en Suisse. Bundesamt für Gesundheit BAG.

[10] (2023). Elderly population (indicator). Organisation for Economic Co-operation and Development.

[11] (2013). Definition of an older or elderly person. World Health Organization.

[12] Sanford AM, Orrell M, Tolson D, Abbatecola AM, Arai H, Bauer JM, Cruz-Jentoft AJ, Dong B, Ga H, Goel A, Hajjar R, Holmerova I, Katz PR, Koopmans RTCM, Rolland Y, Visvanathan R, Woo J, Morley JE, Vellas B (2015). An international definition for "nursing home". J Am Med Dir Assoc.

[13] Nelluri S, Rodriguez-Suarez MM, Rahaman Z, Cabrera K, Priyadarshni S, Pamarthi M, Dang S, Valencia W, Mintzer MJ, Ruiz JG (2018). Coexisting frailty and depression in older veterans: effects on health care utilization. Am J Geriatr Psychiatry.

[14] Ell K (2006). Depression care for the elderly: reducing barriers to evidence-based practice. Home Health Care Serv Q.

[15] Volicer L, Van der Steen JT, Frijters DHM (2009). Modifiable factors related to abusive behaviors in nursing home residents with dementia. J Am Med Dir Assoc.

[16] Gulka HJ, Patel V, Arora T, McArthur C, Iaboni A (2020). Efficacy and generalizability of falls prevention interventions in nursing homes: a systematic review and meta-analysis. J Am Med Dir Assoc.

[17] Levin CA, Wei W, Akincigil A, Lucas JA, Bilder S, Crystal S (2007). Prevalence and treatment of diagnosed depression among elderly nursing home residents in Ohio. J Am Med Dir Assoc.

[18] Boorsma M, Joling K, Dussel M, Ribbe M, Frijters D, van Marwijk HWJ, Nijpels G, van Hout H (2012). The incidence of depression and its risk factors in Dutch nursing homes and residential care homes. Am J Geriatr Psychiatry.

[19] Crogan NL, Evans BC (2008). Quality improvement in nursing homes: identifying depressed residents is critical to improving quality of life. Ariz Geriatr Soc J.

[20] Teresi J, Abrams R, Holmes D, Ramirez M, Eimicke J (2001). Prevalence of depression and depression recognition in nursing homes. Soc Psychiatry Psychiatr Epidemiol.

[21] (2023). StatPearls.

[22] Choi NG, Gell NM, DiNitto DM, Marti CN, Kunik ME (2020). Depression and activity-limiting fall worry among older adults: longitudinal reciprocal relationships. Int Psychogeriatr.

[23] Asp M, Lindqvist D, Fernström Johan, Ambrus L, Tuninger E, Reis M, Westrin Å (2020). Recognition of personality disorder and anxiety disorder comorbidity in patients treated for depression in secondary psychiatric care. PLoS One.

[24] Choi NG, Marti CN, DiNitto DM, Kunik ME (2019). Longitudinal associations of falls and depressive symptoms in older adults. Gerontologist.

[25] Huynh D, Lee ON, An PM, Ens TA, Mannion CA (2021). Bedrails and falls in nursing homes: a systematic review. Clin Nurs Res.

[26] Aprahamian I, Landowski A, Ahn FO, Neves BA, Rocha JT, Strauss J, Borges MK, Morley JE, Oude Voshaar Richard C (2022). Frailty in geriatric psychiatry inpatients: a retrospective cohort study. Int Psychogeriatr.

[27] Rizka A, Indrarespati A, Dwimartutie N, Muhadi M (2021). Frailty among older adults living in nursing homes in Indonesia: prevalence and associated factors. Ann Geriatr Med Res.

[28] Kojima G (2015). Prevalence of frailty in nursing homes: a systematic review and meta-analysis. J Am Med Dir Assoc.

[29] Lee S (2021). Falls associated with indoor and outdoor environmental hazards among community-dwelling older adults between men and women. BMC Geriatr.

[30] Jiang Y, Xia Q, Wang J, Zhou P, Jiang S, Diwan VK, Xu B (2019). Environmental risk factors associated with falls among older people living in long-term aged care facilities: a prospective study. Lancet.

[31] Valipoor S, Pati D, Kazem-Zadeh M, Mihandoust S, Mohammadigorji S (2020). Falls in older adults: a systematic review of literature on interior-scale elements of the built environment. J Aging Environ.

[32] Komisar V, Dojnov A, Yang Y, Shishov N, Chong H, Yu Y, Bercovitz I, Cusimano MD, Becker C, Mackey DC, Robinovitch SN (2022). Injuries from falls by older adults in long-term care captured on video: prevalence of impacts and injuries to body parts. BMC Geriatr.

[33] Imaginário C, Martins T, Araújo F, Rocha M, Machado PP (2021). Risk factors associated with falls among nursing home residents: a case-control study. Port J Public Health.

[34] Kvæl Linda Aimée Hartford, Bergland A, Telenius EW (2017). Associations between physical function and depression in nursing home residents with mild and moderate dementia: a cross-sectional study. BMJ Open.

[35] Visschedijk JHM, Caljouw MAA, Bakkers E, van Balen R, Achterberg WP (2015). Longitudinal follow-up study on fear of falling during and after rehabilitation in skilled nursing facilities. BMC Geriatr.

[36] Tavsanli NG, Turkmen SN (2015). Fear of falling in elderly people living in a nursing home -- perspective from Manisa. J Pak Med Assoc.

[37] Gambaro E, Gramaglia C, Azzolina D, Campani D, Molin AD, Zeppegno P (2022). The complex associations between late life depression, fear of falling and risk of falls. A systematic review and meta-analysis. Ageing Res Rev.

[38] Fuller GF (2000). Falls in the elderly. Am Fam Physician.

[39] Nease DE, Maloin JM (2003). Depression screening: a practical strategy. J Fam Pract.

[40] Iaboni A, Flint AJ (2013). The complex interplay of depression and falls in older adults: a clinical review. Am J Geriatr Psychiatry.

[41] Holtzer R, Friedman R, Lipton RB, Katz M, Xue X, Verghese J (2007). The relationship between specific cognitive functions and falls in aging. Neuropsychology.

[42] Lohman MC, Fallahi A, Mishio Bawa Eric, Wei J, Merchant AT (2023). Social mediators of the association between depression and falls among older adults. J Aging Health.

[43] Lee DCA, Lalor AF, Russell G, Stolwyk R, Brown T, McDermott F, Haines TP (2017). Understanding temporal relationships between depression, falls, and physical activity in a cohort of post-hospitalized older adults - a breakthrough or a conundrum?. Int Psychogeriatr.

[44] Michalak J, Troje NF, Fischer J, Vollmar P, Heidenreich T, Schulte D (2009). Embodiment of sadness and depression--gait patterns associated with dysphoric mood. Psychosom Med.

[45] Giovannini S, Onder G, van der Roest HG, Topinkova E, Gindin J, Cipriani MC, Denkinger MD, Bernabei R, Liperoti R, SHELTER Study Investigators (2020). Use of antidepressant medications among older adults in European long-term care facilities: a cross-sectional analysis from the SHELTER study. BMC Geriatr.

[46] Marcum ZA, Perera S, Thorpe JM, Switzer GE, Castle NG, Strotmeyer ES, Simonsick EM, Ayonayon HN, Phillips CL, Rubin S, Zucker-Levin AR, Bauer DC, Shorr RI, Kang Y, Gray SL, Hanlon JT, Health ABC Study (2016). Antidepressant use and recurrent falls in community-dwelling older adults: findings from the health ABC study. Ann Pharmacother.

[47] van Poelgeest EP, Pronk AC, Rhebergen D, van der Velde N (2021). Depression, antidepressants and fall risk: therapeutic dilemmas-a clinical review. Eur Geriatr Med.

[48] Toronto CE, Toronto CE, Remington R (2020). Overview of the integrative review. A Step-by-Step Guide to Conducting an Integrative Review.

[49] Whittemore R, Knafl K (2005). The integrative review: updated methodology. J Adv Nurs.

[50] Zecevic AA, Salmoni AW, Speechley M, Vandervoort AA (2006). Defining a fall and reasons for falling: comparisons among the views of seniors, health care providers, and the research literature. Gerontologist.

[51] Harrison R, Jones B, Gardner P, Lawton R (2021). Correction to: Quality assessment with diverse studies (QuADS): an appraisal tool for methodological and reporting quality in systematic reviews of mixed- or multimethod studies. BMC Health Serv Res.

[52] Moher D, Liberati A, Tetzlaff J, Altman DG, PRISMA Group (2009). Preferred reporting items for systematic reviews and meta-analyses: the PRISMA statement. BMJ.

[53] Sterne JA, Hernan MA, Reeves BC, Savovic J, Berkman ND, Viswanathan M, Henry D, Altman DG, Ansari MT, Boutron I, Carpenter JR, Chan AW, Churchill R, Deeks JJ, Hróbjartsson A, Kirkham J, Jüni P, Loke YK, Pigott TD, Ramsay CR, Regidor D, Rothstein HR, Sandhu L, Santaguida PL, Schünemann HJ, Shea B, Shrier I, Tugwell P, Turner L, Valentine JC, Waddington H, Waters E, Wells GA, Whiting PF, Higgins JPT (2016). ROBINS-I: a tool for assessing risk of bias in non-randomised studies of interventions. BMJ.

[54] Wells G (2000). The Newcastle-Ottawa Scale (NOS) for assessing the quality of nonrandomised studies in meta-analyses. Ottawa Hospital Research Institute.

[55] Downes MJ, Brennan ML, Williams HC, Dean RS (2016). Development of a critical appraisal tool to assess the quality of cross-sectional studies (AXIS). BMJ Open.

[56] Borenstein M, Higgins JPT (2013). Meta-analysis and subgroups. Prev Sci.

[57] Egger M, Davey Smith G, Schneider M, Minder C (1997). Bias in meta-analysis detected by a simple, graphical test. BMJ.

[58] Higgins JPT, Thompson SG (2002). Quantifying heterogeneity in a meta-analysis. Stat Med.

[59] Menant JC, Wong AKW, Trollor JN, Close JCT, Lord SR (2016). Depressive symptoms and orthostatic hypotension are risk factors for unexplained falls in community-living older people. J Am Geriatr Soc.

[60] Kron M, Loy S, Sturm E, Nikolaus TH, Becker C (2003). Risk indicators for falls in institutionalized frail elderly. Am J Epidemiol.

[61] Wei TS, Liu PT, Chang LW, Liu SY (2017). Gait asymmetry, ankle spasticity, and depression as independent predictors of falls in ambulatory stroke patients. PLoS One.

